# Random-Forest-Based Smartphone GNSS Position Correction Using Satellite-Wise LOS Projection Error Estimation and Exponential Temporal WLS

**DOI:** 10.3390/s26134166

**Published:** 2026-07-02

**Authors:** Kyeongdong Jang, Keonwon Seo

**Affiliations:** Department of Civil Engineering, School of Architectural, Civil, Environmental, and Energy Engineering, Kyungpook National University, Daegu 41566, Republic of Korea; jgd0628@knu.ac.kr

**Keywords:** smartphone GNSS, code pseudorange, random forest, LOS projection error, weighted least squares, temporal WLS, position correction

## Abstract

Smartphone global navigation satellite system (GNSS) positioning is degraded by low-cost antennas, limited receiver hardware, multipath propagation, and noisy code pseudorange observations. Existing correction methods often improve stochastic weighting, estimate coordinate-domain corrections, or smooth receiver trajectories, but they rarely estimate how each satellite contributes to the horizontal position error while preserving line-of-sight (LOS) geometry. This study presents a random-forest-assisted geometry-aware correction method that combines satellite-wise LOS projection error estimation with exponential temporal weighted least squares (Temporal WLS). The horizontal error between the smartphone National Marine Electronics Association (NMEA) solution and the F9P reference position is projected onto each satellite LOS direction and used as the learning target. A random forest model is trained using 26 smartphone GNSS features, including geometry, signal strength, code-derived variation, uncertainty, automatic gain control, and state flags. The predicted LOS errors are fused with satellite geometry through epoch-wise WLS and Temporal WLS. In same-session front-70/back-30 validation, the horizontal root mean square (RMS) error decreased from 2.747 m to 1.033 m. Excluding one suspected non-co-located reference session further reduced the RMS error from 2.867 m to 0.362 m.

## 1. Introduction

Smartphone global navigation satellite system (GNSS) positioning is widely used for vehicle navigation, pedestrian positioning, location-based services, and outdoor augmented-reality applications. Since Android raw GNSS measurements became available, smartphone positioning studies have increasingly used raw code observations, Doppler, carrier phase, carrier-to-noise density ratio ($C/N_{0}$), timing uncertainty, and receiver state information rather than relying only on final NMEA outputs [1,2,3]. Early smartphone positioning studies also demonstrated precise point positioning (PPP) and dual-frequency processing with Android devices, especially using Xiaomi Mi 8 observations [4,5,6]. Public datasets and competitions, particularly the Google Smartphone Decimeter Challenge, have further accelerated research on high-accuracy smartphone GNSS positioning [7,8,9].

However, access to raw measurements does not remove the hardware and propagation limitations of smartphones. Previous studies have reported that smartphone GNSS observations are affected by low-cost antennas, compact receiver hardware, lower signal strength, high code noise, multipath, duty cycling, and device-dependent biases [10,11,12]. Although dual-frequency measurements, accumulated-delta-range (ADR), and carrier-phase observations can support higher precision under favorable conditions, their practical use is often limited by discontinuities, cycle-slip-like behavior, duty cycling, and device-dependent measurement quality [6,13,14,15]. Therefore, code-domain and quality-related features remain important for robust smartphone GNSS correction in practical environments.

One established approach is to improve the stochastic model of smartphone code measurements. Existing methods commonly use $C/N_{0}$, elevation angle, code residual statistics, or combined $C/N_{0}$–elevation weighting to improve weighted least squares (WLS), PPP, or filtering performance [16,17,18]. Abnormal-error detection and correction have also been investigated for smartphone GNSS observations [19]. These methods improve measurement weighting or error screening, but they mainly adjust the observation covariance or detect abnormal measurements rather than learning a continuous satellite-wise position-domain error contribution.

Machine learning has also been used for GNSS multipath and non-line-of-sight (NLOS) detection. Previous studies have formulated the problem as line-of-sight (LOS), multipath, or NLOS classification using correlator outputs, RINEX- or NMEA-level indicators, $C/N_{0}$, residual statistics, clustering, nonlinear regression, or deep learning [20,21,22,23,24]. These methods are useful for signal-quality assessment and multipath mitigation, but they do not directly estimate the continuous contribution of each satellite measurement to the horizontal position error.

Trajectory-level optimization has also been explored, especially in Smartphone Decimeter Challenge studies. Factor graph optimization, GNSS/inertial navigation system integration, velocity constraints, time-differenced carrier phase, and global trajectory optimization have shown strong performance in smartphone trajectory estimation [7,8,9,25,26,27,28]. Learning-based correction methods have also been proposed to improve smartphone positioning by learning coordinate-domain corrections or combining machine learning with filtering frameworks [29,30,31]. However, direct coordinate-domain correction may not explicitly preserve the satellite-receiver geometry that links individual satellite observations to the receiver position.

This study, therefore, formulates the learning problem at the satellite level rather than directly in the coordinate domain. Unlike stochastic weighting methods that only modify observation weights, classification methods that detect multipath or NLOS measurements, and trajectory-optimization methods that directly smooth receiver states, the proposed method learns a continuous satellite-wise LOS projection error target. The horizontal position error is projected onto each satellite’s horizontal LOS direction and used as the satellite-wise learning target. A random forest regressor is trained to estimate this LOS projection error from smartphone raw and NMEA/GSV-derived features. The estimated LOS errors are then fused with satellite geometry through an epoch-wise WLS formulation to reconstruct the horizontal position error. Finally, an exponential Temporal WLS is introduced to jointly estimate a temporally consistent horizontal error using the current and previous epochs, while allowing an epoch-dependent common bias term.

The main contributions of this study are as follows:

1. A satellite-wise LOS projection target is defined by projecting the smartphone horizontal position error onto the horizontal satellite direction, connecting raw satellite-level features with position-domain errors.
2. A random forest estimator is designed using 26 smartphone GNSS quality and geometry features to estimate satellite-wise LOS projection errors.
3. The predicted LOS projection errors are fused with GNSS geometry through epoch-wise WLS and exponential Temporal WLS, rather than being used as a direct coordinate correction or hard satellite-exclusion criterion.
4. The effect of a suspected non-co-located reference session is explicitly reported, demonstrating the importance of co-location between the smartphone and the reference receiver in learning-based smartphone GNSS correction.

## 2. Materials and Methods

### 2.1. Experimental Data and Feature Selection

The smartphone used in this study was a Samsung Galaxy Z Fold5 (SM-F946N; Samsung Electronics Co., Ltd., Suwon, Republic of Korea). Smartphone GNSS measurements were collected using the GNSS/IMU Logger application. The logged data included Android raw GNSS measurements, smartphone NMEA position outputs, and NMEA/GSV satellite information. The reference positions were obtained using a u-blox ZED-F9P receiver (u-blox AG, Thalwil, Switzerland) operated in RTK-fixed mode. In this study, the term RTK-fixed refers only to the F9P reference solution used for supervised label generation and performance evaluation. It does not mean that RTK corrections, ambiguity resolution, or carrier-phase differential processing were applied to the smartphone measurements.

The smartphone log files were transferred to a computer and processed offline in MATLAB R2025b. The processing workflow consisted of Android raw measurement parsing, NMEA/GSV decoding, satellite elevation and azimuth extraction, epoch-level time matching with the F9P reference, feature construction, LOS projection target generation, random-forest-based LOS error estimation, and WLS-based horizontal correction. In the present study, the smartphone was used only for data collection; model training, validation, and WLS/Temporal WLS evaluation were performed offline on a computer. RINEX-based PPP processing, RTKLIB-based smartphone positioning, precise orbit/clock products, smartphone carrier-phase ambiguity resolution, and PPP/RTK corrections were not used in the proposed smartphone correction pipeline.

The data were collected in outdoor field-observation conditions using synchronized smartphone and F9P measurements. The sessions mainly represent open- to semi-open-sky smartphone GNSS observation conditions, with possible local obstructions and multipath from surrounding objects. Dedicated tests under unfavorable weather conditions, dense forest canopy, or deep urban-canyon environments were not performed in the present study. Therefore, the reported performance should be interpreted as validation under the collected same-session field conditions rather than as a complete demonstration across all possible smartphone GNSS environments.

**Figure 1:**
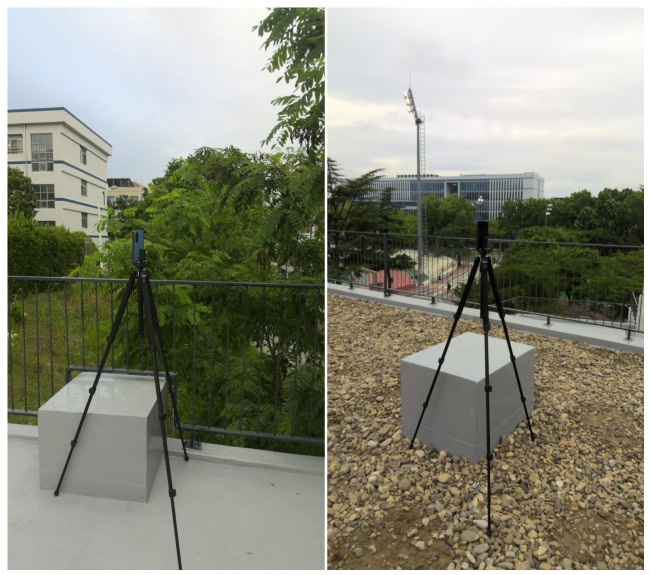
Representative field photographs of the experimental setup used in this study. The left photograph shows the surrounding observation environment, and the right photograph shows the smartphone GNSS measurement setup used together with the reference receiver during data collection. The two images are presented as a single integrated figure to document the observation conditions under which the smartphone and reference data were acquired.

Figure 1 presents representative field photographs of the data-collection campaign. To provide experimental context, the figure shows both the surrounding observation environment and the practical smartphone–reference co-observation setup used for synchronized data acquisition.

For validation, each session was split chronologically into a front 70% segment and a back 30% segment. The front 70% segments from all sessions were then pooled to train a single random forest LOS projection error estimator. The trained RF model was applied to the back 30% segments of all sessions to predict satellite-wise LOS projection errors. These predicted LOS errors were subsequently used in the epoch-wise WLS and Temporal WLS correction steps. Therefore, this validation setting evaluates pooled same-session temporal generalization: the model is trained using earlier portions of the same set of sessions and tested on later portions of those sessions. It should not be interpreted as leave-one-session-out, cross-date, cross-site, or cross-device generalization.

For feature construction, the initial smartphone raw-measurement parser considered approximately 36 candidate variables from Android GNSS raw measurements, NMEA positions, and GSV satellite information. These candidates included satellite geometry, signal-strength indicators, code-derived temporal variation, uncertainty indicators, hardware-related indicators, measurement state flags, and carrier-phase/ADR-related fields. The candidate feature groups and their use in the final model are summarized after the carrier/ADR exclusion analysis.

Accumulated-delta-range (ADR)-related fields were present in the raw smartphone logs, but their state flags did not satisfy the valid carrier-phase condition required for reliable continuous ADR use. In the collected data, the ADR state was mainly reported as 16, which corresponds to a half-cycle-related state and does not include the ADR_STATE_VALID bit. Therefore, ADR values were not treated as valid continuous carrier-phase observations and were excluded from the final model. The final 26-feature model was intentionally restricted to code-domain, geometry, uncertainty, receiver-quality, and epoch-context features. This design avoids dependence on carrier-phase ambiguity resolution and makes the proposed correction framework applicable to smartphone logs in which continuous carrier-phase tracking is not valid or not reliable. Table 1 summarizes how carrier-phase- and ADR-related candidate variables were excluded.

To verify whether carrier-phase-related measurements could be used, a parser-level availability and ADR-state validity scan was performed. The carrier-phase, carrier-cycle, and carrier-phase-uncertainty fields had non-missing ratios of 0.000. For the ADR state field, Table 2 shows that the field was almost always present, but the ADR_STATE_VALID bit ratio was 0.000, while the state-16 ratio was 1.000.

Therefore, ADR- and carrier-phase-related measurements were not treated as valid continuous carrier-phase observations and were excluded from the final 26-feature model. The frequency-related feature retained in the final model represents only the GNSS signal frequency or frequency-band context, not carrier-phase, carrier-cycle, or ADR information.

This design choice differentiates the proposed method from RTK- or PPP-oriented methods. The RTK-fixed solution was used only as the F9P reference trajectory for training-label generation and validation. The proposed smartphone correction method itself does not use ADR continuity, resolve carrier-phase ambiguities, apply differential carrier-phase processing, or construct precise carrier-phase residuals. PPP is mentioned in this paper only as a representative class of high-precision GNSS methods in the literature. No PPP solution was generated or evaluated in this study, and no precise orbit or clock products were used. Instead, the proposed method learns satellite-wise LOS projection errors from smartphone-observable quality features and reconstructs the horizontal position error using WLS.

Table 3 summarizes the candidate feature groups and their use in the final model.

Although pseudorange-rate and pseudorange-related quantities are available or derivable from the Android raw measurements, they were not used as direct final RF predictors in this study. The raw pseudorange-rate measurement was used only as an intermediate quantity for constructing code-derived temporal variation features, whereas the pseudorange-rate uncertainty was retained as a final quality-related feature. Similarly, absolute code pseudorange values can be derived from Android timing fields, but they were not used to construct full GNSS observation equations or precise pseudorange residuals.

### 2.2. LOS Projection Error Learning and Temporal WLS

Table 4 summarizes the main symbols used in the LOS projection and Temporal WLS formulation.

The horizontal position-error target was first defined in the local East–North plane. At epoch *k*, the smartphone NMEA position and the F9P-derived reference position are denoted by

(1) $$P_{k}^{\mathrm{phone}}=\left({\mathrm{lat}}_{k}^{\mathrm{phone}},{\mathrm{lon}}_{k}^{\mathrm{phone}}\right),$$

and

(2) $$P_{k}^{\mathrm{ref}}=\left({\mathrm{lat}}_{k}^{\mathrm{ref}},{\mathrm{lon}}_{k}^{\mathrm{ref}}\right),$$

respectively. The latitude and longitude differences were converted to local East–North errors using a local tangent-plane approximation. In this approximation, one degree of latitude is assumed to correspond to approximately 111,320 m, and the longitude scale is additionally multiplied by the cosine of the reference latitude. This approximation is valid because the horizontal position differences between the smartphone and the reference receiver are small compared with the Earth’s radius.

(3) $$e_{E,k}=\left({\mathrm{lon}}_{k}^{\mathrm{phone}}-{\mathrm{lon}}_{k}^{\mathrm{ref}}\right)\cdot 111320\cdot \cos\left(\frac{\pi }{180}{\mathrm{lat}}_{k}^{\mathrm{ref}}\right),$$

(4) $$e_{N,k}=\left({\mathrm{lat}}_{k}^{\mathrm{phone}}-{\mathrm{lat}}_{k}^{\mathrm{ref}}\right)\cdot 111320.$$

Thus, the raw horizontal position error vector is defined as

(5) $$e_{k}^{\mathrm{raw}}=e_{k}=\left[\begin{array}{c} e_{E,k}\\ e_{N,k} \end{array}\right].$$

The satellite-wise learning target was then constructed by projecting this horizontal error onto the satellite LOS direction. For satellite *i* observed at epoch *k*, the elevation and azimuth angles obtained from GSV information are denoted by ${\mathrm{El}}_{k,i}$ and ${\mathrm{Az}}_{k,i}$, respectively. Because these angles are provided in degrees, they are converted to radians inside the trigonometric functions. The horizontal line-of-sight (LOS) direction components are defined as

(6) $$u_{E,k,i}=\cos\left(\frac{\pi }{180}{\mathrm{El}}_{k,i}\right)\sin\left(\frac{\pi }{180}{\mathrm{Az}}_{k,i}\right),$$

(7) $$u_{N,k,i}=\cos\left(\frac{\pi }{180}{\mathrm{El}}_{k,i}\right)\cos\left(\frac{\pi }{180}{\mathrm{Az}}_{k,i}\right).$$

The horizontal LOS vector is

(8) $$u_{k,i}=\left[\begin{array}{c} u_{E,k,i}\\ u_{N,k,i} \end{array}\right].$$

The satellite-wise learning target is defined by projecting the raw horizontal position error onto the negative LOS direction

(9) $$y_{k,i}=-u_{k,i}^{T}e_{k}^{\mathrm{raw}}.$$

Equivalently,

(10) $$y_{k,i}=-\left(u_{E,k,i}e_{E,k}+u_{N,k,i}e_{N,k}\right).$$

This target represents how the raw horizontal position error is observed along the horizontal satellite direction.

**Figure 2:**
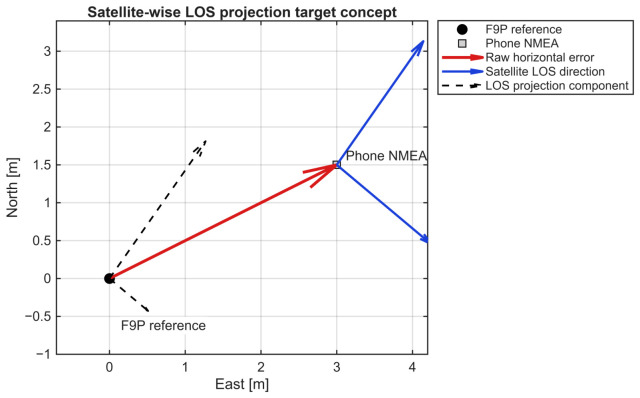
Definition of the satellite-wise LOS projection error target. The raw horizontal NMEA error vector is projected onto the negative horizontal LOS direction of each satellite. This converts a two-dimensional position-domain error into satellite-wise scalar learning targets that can be estimated from smartphone GNSS quality and geometry features.

Figure 2 illustrates this target definition. The projection converts a two-dimensional position-domain error into scalar satellite-wise learning targets while preserving the relation between the error vector and satellite geometry.

Using this satellite-wise target, a random forest model was trained to estimate LOS projection errors from smartphone GNSS features. For each satellite sample, a 26-dimensional feature vector is constructed

(11) $$x_{k,i}={\left[{\mathrm{El}}_{k,i},{\mathrm{Az}}_{k,i},u_{E,k,i},u_{N,k,i},{\left(C/N_{0}\right)}_{k,i},\Delta {\left(C/N_{0}\right)}_{k,i},{\mathrm{relCDD}}_{k,i},\left|{\mathrm{relCDD}}_{k,i}\right|,\ldots \right]}^{T}.$$

The feature set includes satellite geometry, signal strength, code-derived relative variation, uncertainty measures, automatic gain control, and GNSS state indicators.

The final 26 input features are listed in Table 5. The feature set intentionally combines weak but complementary indicators rather than relying on a single signal-strength metric. The frequency-context feature should not be interpreted as carrier-phase information.

The uncertainty-related features in Table 5 were derived from Android raw GNSS measurement fields. The SV time uncertainty represents the receiver-reported uncertainty of the received satellite time, and the pseudorange-rate uncertainty represents the receiver-reported uncertainty of the pseudorange-rate measurement. These quantities were used as quality indicators rather than as direct observation residuals. Log-transformed versions were also included to reduce scale imbalance and allow the random forest model to use both the original and compressed uncertainty scales. In the MATLAB preprocessing workflow, non-finite or unavailable values were replaced with predefined neutral values before model training and prediction. The raw pseudorange-rate measurement itself was used only as an intermediate quantity for constructing code-derived temporal variation features, whereas its uncertainty was retained as a final input feature.

A random forest estimator is trained to predict the satellite-wise LOS projection error

(12) $${\hat{y}}_{k,i}=\hat{f}\left(x_{k,i}\right).$$

The supervised regression objective can be interpreted as minimizing the squared prediction error [32]

(13) $$\hat{f}=\arg\min_{f}\sum_{k=1}^{N_{\mathrm{tr}}}\sum_{i=1}^{m_{k}}{\left[y_{k,i}-f\left(x_{k,i}\right)\right]}^{2},$$

where $N_{\mathrm{tr}}$ is the number of training epochs, $m_{k}$ is the number of satellites observed at epoch *k*, and $\hat{f}$ denotes the trained random forest predictor.

In this study, the random forest estimator was implemented using 500 trees, a minimum leaf size of 1, and all available predictors considered at each split.

**Figure 3:**
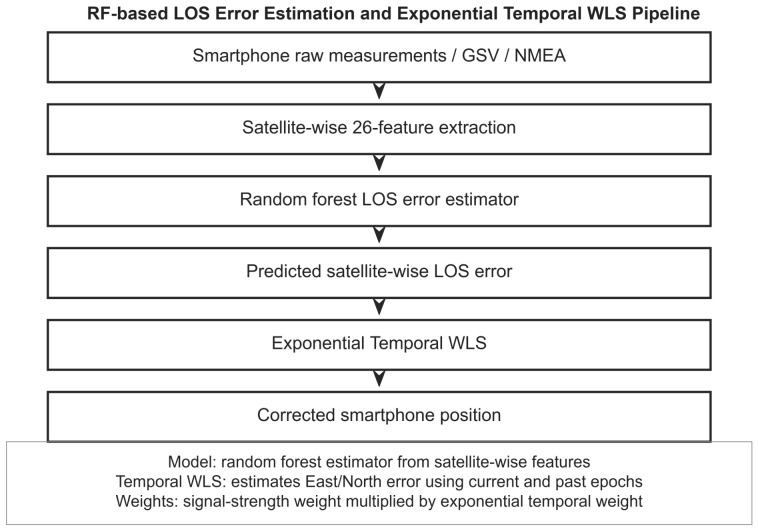
Overall processing pipeline of the proposed smartphone GNSS correction method. Smartphone raw measurements, NMEA positions, and GSV satellite information are used to construct satellite-wise input features. The horizontal NMEA error relative to the F9P reference is projected onto each satellite LOS direction to form the learning target. A random forest estimator predicts the satellite-wise LOS projection error, and the predicted errors are fused with satellite geometry through epoch-wise WLS and exponential Temporal WLS.

Figure 3 summarizes the overall processing pipeline from smartphone GNSS feature extraction to geometry-aware temporal correction.

The predicted LOS projection errors were first converted into a horizontal position correction using an epoch-wise WLS formulation. At epoch *k*, let $m_{k}$ be the number of satellites used in the solution. The predicted LOS projection error vector is

(14) $${\hat{y}}_{k}={\left[\begin{array}{cccc} {\hat{y}}_{k,1} & {\hat{y}}_{k,2} & \cdots & {\hat{y}}_{k,m_{k}} \end{array}\right]}^{T}.$$

For each satellite, the observation equation is expressed as

(15) $${\hat{y}}_{k,i}=-u_{E,k,i}\Delta E_{k}-u_{N,k,i}\Delta N_{k}+b_{k}+\epsilon_{k,i},$$

where $\Delta E_{k}$ and $\Delta N_{k}$ are the horizontal position error components to be estimated, $b_{k}$ is an epoch-wise common bias, and $\epsilon_{k,i}$ is the residual.

In matrix form,

(16) $${\hat{y}}_{k}=H_{k}z_{k}+\epsilon_{k},$$

where the state vector and design matrix are

(17) $$z_{k}={\left[\begin{array}{ccc} \Delta E_{k} & \Delta N_{k} & b_{k} \end{array}\right]}^{T},$$

and

(18) $$H_{k}=\left[\begin{array}{ccc} -u_{E,k,1} & -u_{N,k,1} & 1\\ -u_{E,k,2} & -u_{N,k,2} & 1\\ \vdots & \vdots & \vdots \\ -u_{E,k,m_{k}} & -u_{N,k,m_{k}} & 1 \end{array}\right].$$

The satellite weight is based on $C/N_{0}$

(19) $$w_{k,i}=10^{{\left(C/N_{0}\right)}_{k,i}/10}.$$

The corresponding weight matrix is

(20) $$W_{k}=\mathrm{diag}\left(w_{k,1},w_{k,2},\ldots ,w_{k,m_{k}}\right).$$

The weighted least-squares (WLS) solution is obtained as

(21) $${\hat{z}}_{k}={\left(H_{k}^{T}W_{k}H_{k}\right)}^{-1}H_{k}^{T}W_{k}{\hat{y}}_{k}.$$

The first two components of ${\hat{z}}_{k}$ yield the estimated horizontal error

(22) $${\hat{e}}_{k}={\left[\begin{array}{cc} {\hat{\Delta E}}_{k} & {\hat{\Delta N}}_{k} \end{array}\right]}^{T}.$$

Because $e_{k}^{\mathrm{raw}}$ denotes the raw NMEA error relative to the reference position, the corrected residual error is computed as

(23) $$e_{k}^{\mathrm{corr}}=e_{k}^{\mathrm{raw}}-{\hat{e}}_{k}.$$

To reduce epoch-wise prediction noise, the correction was further stabilized using exponential Temporal WLS. Because the RF-predicted LOS errors contain instantaneous prediction noise, the epoch-wise WLS solution can fluctuate. The prediction model can be expressed as

(24) $${\hat{y}}_{k,i}=y_{k,i}+\eta_{k,i},$$

where $\eta_{k,i}$ represents the prediction residual.

To reduce this noise, the proposed method jointly estimates a temporally consistent horizontal error using a causal window. For the current epoch *K*, the temporal window is defined as

(25) $$S_{K}=\left\{K-M+1,K-M+2,\ldots ,K\right\}.$$

The final configuration uses M=480 epochs. The exponential temporal weight for epoch $j\in S_{K}$ is

(26) $$\alpha_{j}=\exp\left(-\frac{K-j}{\tau }\right),$$

with τ=720 epochs.

Within this localized temporal window, the horizontal error is assumed to vary slowly

(27) $$e_{j}^{\mathrm{raw}}\approx e_{K}^{\mathrm{raw}},\;j\in S_{K}.$$

Thus,

(28) $$\begin{array}{cc} e_{E,j} & \approx E_{0}, \end{array}$$

(29) $$\begin{array}{cc} e_{N,j} & \approx N_{0}. \end{array}$$

Here, $E_{0}$ and $N_{0}$ denote the common horizontal error components estimated for the current temporal window, not absolute coordinates. The common bias term is estimated separately for each epoch to absorb epoch-dependent offsets in the predicted LOS errors. The temporal observation equation is written as

(30) $${\hat{y}}_{j,i}=-u_{E,j,i}E_{0}-u_{N,j,i}N_{0}+b_{j}+\epsilon_{j,i}.$$

Assume that the causal window contains *L* unique epochs, $S_{K}=\left\{j_{1},j_{2},\ldots ,j_{L}\right\}$. The joint parameter vector is

(31) $$q_{K}={\left[\begin{array}{cccccc} E_{0} & N_{0} & b_{j_{1}} & b_{j_{2}} & \cdots & b_{j_{L}} \end{array}\right]}^{T}.$$

Let ℓ(j) denote the local index of epoch *j* within the window, i.e., $j=j_{\ell (j)}$. The design row corresponding to satellite *i* at epoch *j* is

(32) $$a_{j,i}=\left[\begin{array}{ccccc} -u_{E,j,i} & -u_{N,j,i} & 0_{1\times (\ell (j)-1)} & 1 & 0_{1\times (L-\ell (j))} \end{array}\right].$$

The stacked temporal system is

(33) $${\hat{y}}_{S}=A_{S}q_{K}+\epsilon_{S}.$$

The final observation weight is the product of the signal-strength weight and the exponential temporal weight

(34) $$w_{j,i}=10^{{\left(C/N_{0}\right)}_{j,i}/10}\cdot \exp\left(-\frac{K-j}{\tau }\right).$$

The temporal weight matrix is defined as

(35) $$W_{S}=\mathrm{diag}\left(w_{j,i}\right),\;j\in S_{K}.$$

The temporal WLS solution is obtained as

(36) $${\hat{q}}_{K}={\left(A_{S}^{T}W_{S}A_{S}\right)}^{-1}A_{S}^{T}W_{S}{\hat{y}}_{S}.$$

The final horizontal error estimate for the current epoch is

(37) $${\hat{e}}_{K}={\left[\begin{array}{cc} {\hat{E}}_{0} & {\hat{N}}_{0} \end{array}\right]}^{T}.$$

The corrected residual error for the current epoch is therefore

(38) $$e_{K}^{\mathrm{corr}}=e_{K}^{\mathrm{raw}}-{\hat{e}}_{K}.$$

The equivalent optimization problem for the final configuration is written as [33]

(39) $$\min_{E,N,\{b_{j}\}}\sum_{j=K-479}^{K}\sum_{i}10^{{\left(C/N_{0}\right)}_{j,i}/10}\exp\left(-\frac{K-j}{720}\right){\left[{\hat{y}}_{j,i}+u_{E,j,i}E+u_{N,j,i}N-b_{j}\right]}^{2}.$$

The proposed method assumes that the smartphone and the F9P reference receiver are co-located during data collection. If a physical offset exists between the smartphone and the reference antenna, the observed label becomes

(40) $$e_{k}^{\mathrm{label}}=e_{k}^{\mathrm{gnss}}+d,$$

where $d={\left[d_{E},d_{N}\right]}^{T}$ is the physical displacement between the two receivers. The corresponding target becomes

(41) $$y_{k,i}^{\mathrm{label}}=-u_{k,i}^{T}\left(e_{k}^{\mathrm{gnss}}+d\right)$$

(42) $$=-u_{k,i}^{T}e_{k}^{\mathrm{gnss}}-u_{k,i}^{T}d.$$

The second term is not caused by GNSS signal quality but by receiver placement. Therefore, a session suspected of non-co-located observation was separately reported in the results.

The positioning performance was evaluated using the two-dimensional root-mean-square (RMS) error, defined as

(43) $$\mathrm{RMS}=\sqrt{\frac{1}{N}\sum_{k=1}^{N}\left[{\left(e_{E,k}^{\mathrm{corr}}\right)}^{2}+{\left(e_{N,k}^{\mathrm{corr}}\right)}^{2}\right]}.$$

The raw RMS error is obtained by replacing the corrected components with the raw NMEA error components.

## 3. Results

### 3.1. Feature and LOS Projection Error Prediction Analysis

Before selecting the final correction architecture, the relationship between the selected input features and the LOS projection target *y* was analyzed. The single-feature linear correlation analysis showed that no individual feature had a strong linear relationship with the target. The strongest representative correlations were observed for SV time uncertainty, $C/N_{0}$, and pseudorange-rate uncertainty, but their magnitudes were only approximately 0.1. In particular, signal-strength-related features such as $C/N_{0}$ and $\Delta C/N_{0}$ were insufficient to explain the LOS projection target by themselves. Therefore, simple signal-strength weighting or single-feature correction cannot adequately estimate *y*.

In contrast, the RF-based feature-importance analysis indicated that geometry-related and quality-related features jointly contributed to nonlinear prediction. Elevation angle, AGC, pseudorange-rate uncertainty, relative code-derived variation, measurement state flags, $C/N_{0}$, and time uncertainty appeared as influential features. This supports the use of a nonlinear ensemble estimator that combines multiple weakly informative indicators.

**Figure 4:**
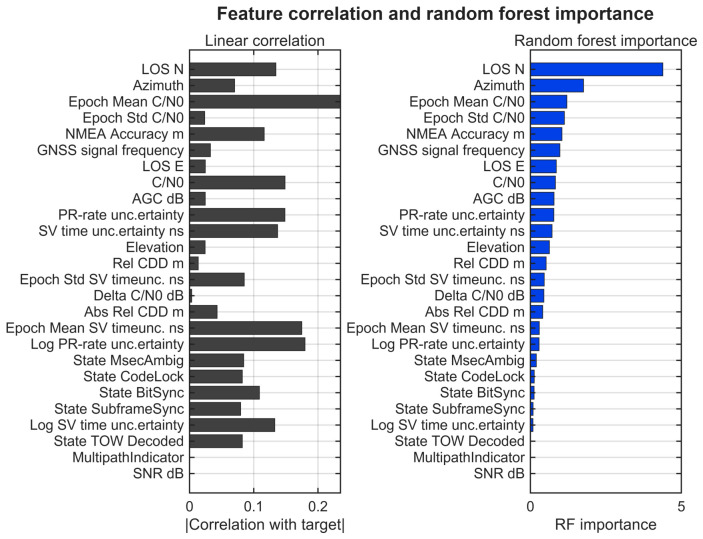
Feature analysis for the 26 selected smartphone GNSS input features. The left panel shows absolute single-feature linear correlation with the LOS projection target. The right panel shows MATLAB out-of-bag permuted predictor importance from the random forest model. The RF importance values are relative indicators of contribution to prediction accuracy and do not have physical units. The weak linear correlations and distributed RF importance indicate that the LOS projection error is estimated through a nonlinear combination of multiple weak quality and geometry indicators rather than through a single dominant feature.

Table 6 summarizes the feature-analysis results for the 26 selected input features. Figure 4 visualizes the contrast between weak single-feature linear correlation and RF-based nonlinear importance.

Figure 4 visualizes the feature-analysis result. The left panel shows the absolute linear correlation between each feature and the LOS projection target. The weak single-feature correlations indicate that no individual indicator, such as signal strength or code-derived variation alone, can reliably estimate the target. The right panel shows the random forest predictor importance computed using MATLAB’s out-of-bag permuted predictor importance. These values represent relative changes in out-of-bag prediction error when each predictor is permuted; they are therefore relative importance indicators rather than physical units. The result suggests that the RF estimator uses nonlinear combinations of geometry, signal context, NMEA accuracy, uncertainty, and code-derived features. However, because the validation is based on a same-session front-70/back-30 split, the reported prediction performance should be interpreted together with possible same-session temporal dependence.

For the LOS projection error estimation task, the random forest estimator achieved a validation mean absolute error of 0.445 m, an RMSE of 0.689 m, and a correlation coefficient of 0.884 between the predicted and target LOS projection errors in the back 30% validation data. The reported validation metrics were computed on the pooled back 30% validation segments using the single RF model trained from the pooled front 70% training segments. These results indicate that the satellite-wise feature set contains information related to the projected horizontal position error, although the predicted LOS values still include instantaneous noise.

### 3.2. Temporal Parameter Selection and Positioning Performance

The Temporal WLS parameters were selected by evaluating the smoothing behavior of the exponential window. The Temporal WLS formulation in Equation (39) contains two smoothing parameters: the exponential forgetting constant τ and the maximum causal window length *M*. The maximum window length *M* determines the number of causal epochs included in the joint WLS estimation, whereas τ controls how rapidly the contribution of older epochs decays through the temporal weight exp[−(K−j)/τ]. A smaller τ emphasizes recent epochs more strongly, while a larger τ retains stronger contributions from older epochs within the same causal window.

Table 7 summarizes the tested τ–*M* grid using all-data, suspect-session-excluded, and session-aggregated RMS values. The purpose of this table is to show the stability of the Temporal WLS behavior over a range of causal smoothing parameters rather than to claim that the method is strongly dependent on one specific pair of values. The all-data RMS was relatively insensitive to the tested parameters once a sufficiently long causal window was used. The lowest all-data RMS was obtained with τ=240 epochs and M=480 epochs. However, this all-data criterion includes the suspected non-co-located reference session. When the suspected session was excluded, τ=720 epochs and M=480 epochs produced the lowest RMS. The same setting also produced the lowest mean and median per-session RMS among the tested configurations. Therefore, τ=720 epochs and M=480 epochs were selected as the final Temporal WLS configuration. With this setting, the oldest epoch in the causal window retains a temporal weight of approximately exp(−479/720)=0.514 relative to the current epoch.

Table 8 summarizes the overall performance after applying the selected τ=720, M=480 Temporal WLS configuration. For all validation sessions, the raw smartphone NMEA RMS error was 2.747 m. The epoch-wise RF y-WLS reduced the RMS error to 1.109 m, and the proposed exponential Temporal WLS further reduced it to 1.033 m. When the suspected non-co-located reference session was excluded, the raw NMEA RMS error was 2.867 m, while the proposed method reduced the RMS error to 0.362 m.

**Figure 5:**
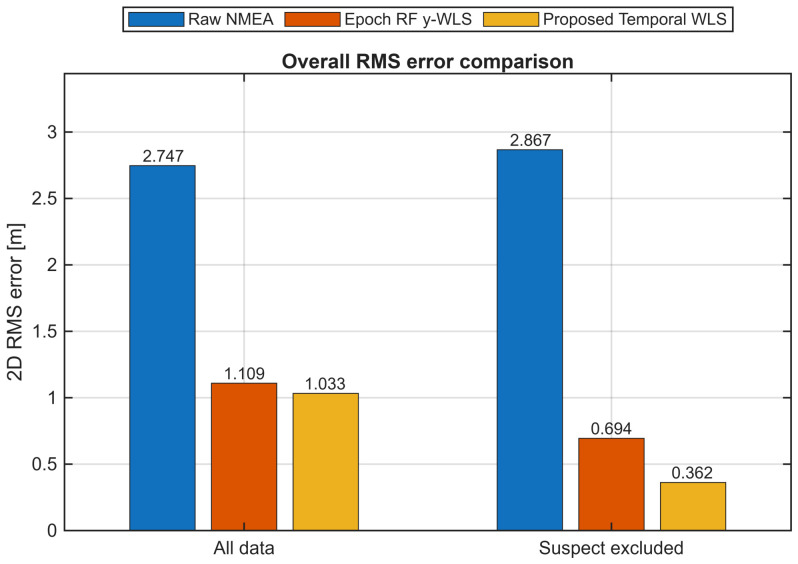
Overall horizontal RMS error comparison. The proposed RF-Temporal WLS method reduces the error of the raw smartphone NMEA solution by combining satellite-wise LOS error estimation with geometry-aware temporal smoothing. Results are shown for all validation sessions and for the subset excluding the suspected non-co-located reference session.

Figure 5 visualizes the overall RMS reduction. The largest improvement is obtained after applying the proposed Temporal WLS, especially when the suspected non-co-located reference session is excluded.

Table 9 shows the per-session RMS errors. Five of the six validation sessions improved from the epoch-wise RF y-WLS to the proposed Temporal WLS. Session S4 was flagged as a suspected non-co-located reference session because the Temporal WLS result was slightly worse than the epoch-wise result and the session behaved differently from the other sessions.

**Figure 6:**
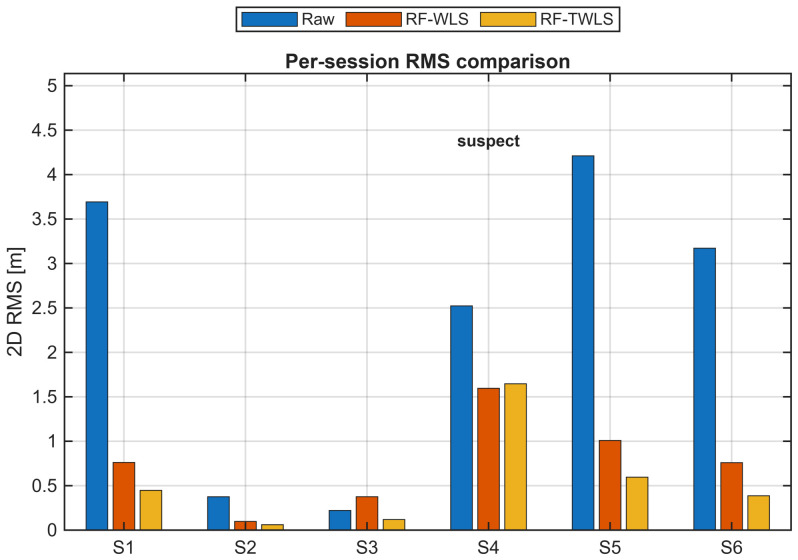
Per-session horizontal RMS error comparison for raw NMEA, epoch-wise RF y-WLS, and RF-Temporal WLS. Most sessions show consistent improvement after temporal smoothing, whereas the session flagged as a suspected non-co-located reference case is reported separately.

**Figure 7:**
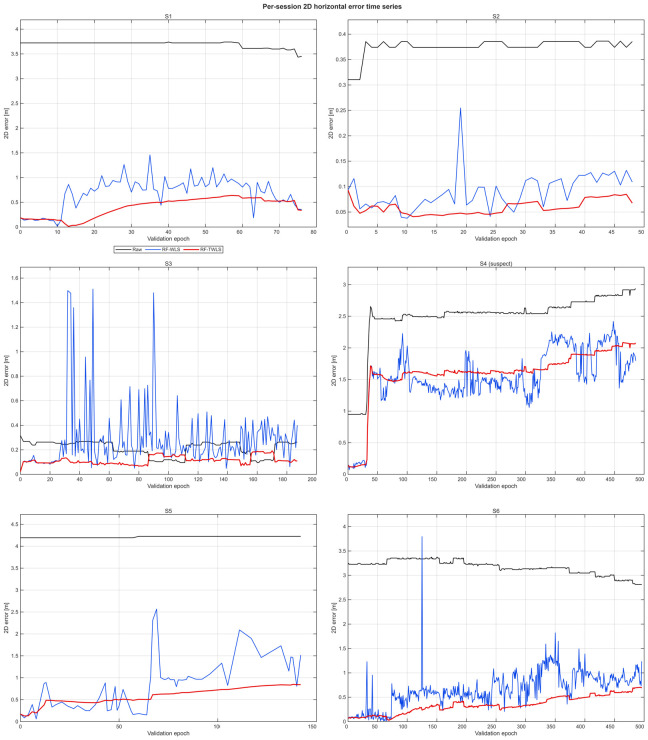
Per-session time-series comparison of two-dimensional horizontal error. The proposed RF-Temporal WLS suppresses short-term fluctuations in the epoch-wise RF y-WLS solution and provides smoother error trajectories over the validation period.

**Figure 8:**
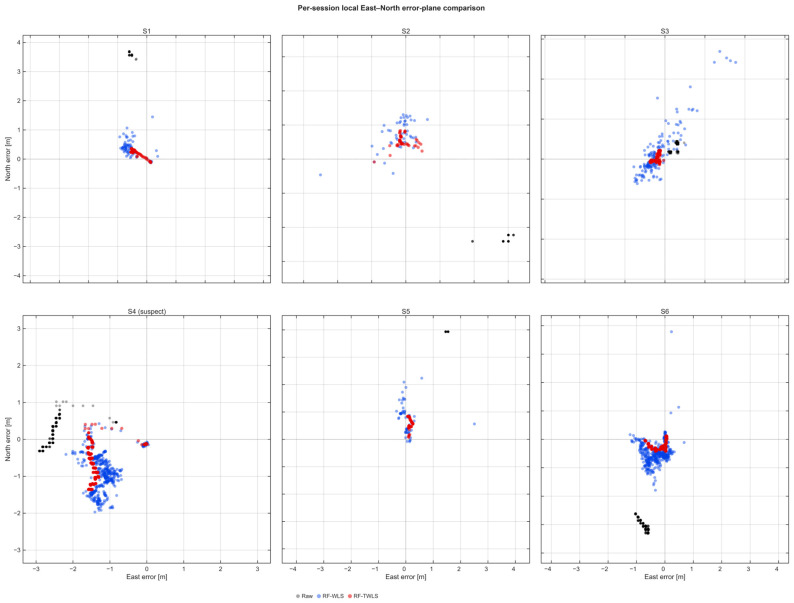
Per-session local East–North error-plane comparison. Black, blue, and red points represent the raw NMEA solution, epoch-wise RF y-WLS solution, and RF-Temporal WLS solution, respectively. The origin (0,0) corresponds to the F9P reference position. The concentration of red points near the origin indicates the effect of the proposed temporal geometry-aware correction.

Figure 6 compares the RMS error for each validation session. Figure 7 further shows the temporal behavior of the two-dimensional error, and Figure 8 provides a spatial interpretation in the local East–North error plane.

## 4. Discussion

The results show that the proposed LOS projection target provides a useful link between satellite-wise smartphone GNSS features and position-domain correction. The epoch-wise RF y-WLS reduced the all-data RMS error from 2.747 m to 1.109 m, confirming that the predicted LOS projection errors can reconstruct a meaningful horizontal correction through WLS. The exponential Temporal WLS further reduced short-term fluctuations and produced an all-data RMS error of 1.033 m.

Recent smartphone GNSS studies have advanced mainly in three directions. First, stochastic-model studies improve code-observation weighting using indicators such as elevation angle, $C/N_{0}$, and measurement uncertainty [16,17,18]. Second, recent Smartphone Decimeter Challenge studies have emphasized trajectory-level optimization, GNSS/IMU factor-graph processing, velocity constraints, and carrier-phase- or TDCP-based smoothing [26,27,28]. Third, learning-based approaches have been used to estimate coordinate-domain or PVT-domain corrections and to combine machine learning with filtering frameworks [29,30,31]. Compared with these recent developments, the proposed method is positioned between measurement-quality modeling and coordinate-domain correction. It estimates satellite-wise position-domain LOS projection errors from smartphone-observable features and then reconstructs the horizontal correction through geometry-aware WLS. Therefore, the contribution is methodological and complementary rather than a claim of universal performance superiority over all existing smartphone GNSS correction methods.

The small RMS values should be interpreted within the same-session front-70/back-30 validation setting. Because the training and validation data are different temporal portions of the same sessions, they can share receiver behavior, satellite visibility, NMEA smoothing characteristics, and environmental conditions. The suspect-session-excluded RMS of 0.362 m is therefore reported as a sensitivity result, and the 0.061 m value corresponds to the best individual normal session rather than the overall representative performance. These results demonstrate the potential of the LOS-geometry-based framework under same-session temporal validation, but they should not be interpreted as proof of centimeter-level generalization across unseen sessions, different dates, different environments, or different smartphone models.

One session was flagged as a suspected non-co-located reference case. This flag was not assigned solely because the session had a larger error; rather, the field-observation record and the distinct per-session behavior suggested a possible physical offset between the F9P reference observation point and the smartphone observation point. If such an offset exists, the generated label contains both the smartphone GNSS positioning error and the receiver-placement displacement. This displacement is unrelated to satellite signal quality, code noise, or multipath, and cannot be learned reliably from the smartphone GNSS feature set. For this reason, the all-data result is reported as the primary result, while the suspect-session-excluded result is reported separately.

The session-to-session variation also suggests that correction quality may be predictable from session-level GNSS quality indicators. Candidate indicators include satellite count, $C/N_{0}$ distribution, elevation distribution, NMEA-reported accuracy, predicted LOS-error stability, WLS residuals, and the conditioning of the WLS design matrix. Such indicators could support a future quality-aware classifier that selects between raw NMEA, epoch-wise RF y-WLS, and RF-Temporal WLS, or flags sessions that are unsuitable for temporal smoothing.

This study has several limitations. The validation was performed using a front-70/back-30 split within the same sessions, and further leave-one-session-out, cross-date, and cross-environment validation is required. The experiments used a single smartphone model and did not include dedicated adverse-weather, dense-forest, or deep urban-canyon tests. The method also requires F9P reference positions during supervised training, although the correction phase uses only smartphone-observable features after training. Finally, the proposed algorithm was evaluated offline in MATLAB; real-time smartphone or edge-device implementation should be examined in future work.

## 5. Conclusions

This study proposed a smartphone GNSS position correction method that combines a random-forest-based satellite-wise LOS projection error estimator with an exponential Temporal WLS. The proposed method first estimates a satellite-wise projected error from smartphone GNSS quality and geometry features, then reconstructs the horizontal position error using a geometry-aware WLS formulation. Temporal smoothing using an exponential window further stabilizes the solution.

In same-session front-70/back-30 validation, the proposed method reduced the all-data horizontal RMS error from 2.747 m to 1.033 m. When a suspected non-co-located reference session was excluded in a sensitivity analysis, the RMS error was reduced from 2.867 m to 0.362 m, and the lowest per-session RMS among the normal sessions was 0.061 m. These findings show that satellite-wise quality features and LOS geometry can be combined effectively for smartphone GNSS position correction within the same-session temporal validation setting. The results also indicate that the proposed method does not require external RTK or PPP corrections during the smartphone correction phase. Future work will focus on broader generalization testing, including models trained on multiple sessions and applied to unseen sessions, different dates, different environments, and additional smartphone models without reference input during correction. In particular, adverse-weather, forested, and urban-canyon environments should be evaluated to clarify the robustness of the proposed correction framework. Another important direction is real-time implementation, where the trained model and WLS/Temporal WLS correction module are deployed on a smartphone or edge device to assess computational latency, memory use, and power consumption. These studies will be combined with adaptive temporal modeling, quality-aware session classification, and broader comparison with existing stochastic-model and machine-learning-based smartphone GNSS correction methods.

## Figures and Tables

**Table 1:** Exclusion of carrier-phase- and ADR-related candidate measurements.

Candidate Group	Example Fields	Used	Reason for Exclusion
Accumulated delta range (ADR)	ADR value, ADR uncertainty	No	No valid continuous ADR state.
ADR state indicators	ADR valid, reset, cycle-slip, half-cycle state bits	No	State 16; VALID bit not set.
Carrier-phase fields	Carrier phase, carrier cycles, carrier-phase uncertainty	No	No valid ADR continuity.
Carrier-phase ambiguity	Integer ambiguity-related information	No	No ambiguity resolution or carrier-phase residual modeling.
Absolute raw pseudorange	Absolute code pseudorange	No	No precise absolute pseudorange residual modeling.
Code-domain temporal variation	Relative CDD, absolute relative CDD	Yes	Code-domain temporal variation without ADR continuity.

**Table 2:** Parser-level ADR state validity summary.

Field Group	Non-Missing	VALID Bit	RESET Bit	Cycle-Slip Bit	State-16
ADR state	0.99999	0.000	0.000	0.000	1.000

**Table 3:** Candidate feature groups and final selection.

Category	Candidate Feature	Used	Reason
Geometry	Elevation, azimuth	Yes	LOS geometry
Geometry	LOS East/North components	Yes	WLS geometry
Signal strength	$C/N_{0}$, $\Delta C/N_{0}$	Yes	Signal quality
Code-derived	Relative CDD, absolute relative CDD	Yes	Code variation
Uncertainty	SV time uncertainty, log SV time uncertainty	Yes	Timing quality
Uncertainty	Pseudorange-rate uncertainty, log uncertainty	Yes	Rate uncertainty
Hardware	AGC	Yes	Receiver gain state
State	Code-lock, bit-sync, subframe-sync, TOW-decoded, millisecond ambiguity flags	Yes	Measurement state
Quality indicator	Multipath indicator, SNR	Yes	Quality indicator
Frequency/NMEA	GNSS signal frequency, NMEA accuracy	Yes	Frequency/NMEA context
Epoch context	Mean/standard deviation of $C/N_{0}$ and SV time uncertainty	Yes	Epoch context
Carrier/ADR	Accumulated delta range, ADR uncertainty, ADR state	No	Invalid/unstable ADR
Carrier phase	Carrier phase, carrier cycles, carrier-phase uncertainty	No	No valid carrier-phase use
Raw Doppler	Pseudorange-rate measurement	No	Not a direct RF input
Raw pseudorange	Absolute code pseudorange	No	No absolute residual model

**Table 4:** Main notation used in the LOS projection error and Temporal WLS formulation.

Symbol	Meaning
*k* or *K*	Epoch index; *K* denotes the current epoch in Temporal WLS.
*i*	Satellite index at a given epoch.
$e_{k}^{\mathrm{raw}}$	Raw horizontal NMEA error vector relative to the F9P reference at epoch *k*.
$e_{E,k},e_{N,k}$	East and North components of the raw horizontal error.
$u_{k,i}$	Horizontal LOS unit vector of satellite *i* at epoch *k*.
$u_{E,k,i},u_{N,k,i}$	East and North components of the horizontal LOS direction.
$y_{k,i}$	Supervised satellite-wise LOS projection error target.
${\hat{y}}_{k,i}$	Random-forest-predicted LOS projection error.
$b_{k}$	Epoch-wise common bias term in epoch-wise WLS.
$S_{K}$	Causal temporal window used for the current epoch *K*.
*M*	Maximum number of causal epochs in Temporal WLS.
τ	Exponential forgetting constant in Temporal WLS.
$E_{0},N_{0}$	Common East and North horizontal error components estimated within the temporal window.
$b_{j}$	Epoch-dependent common bias term for epoch *j* inside the temporal window.
${\hat{e}}_{k}$ or ${\hat{e}}_{K}$	Estimated horizontal error vector reconstructed by WLS or Temporal WLS.
$e_{k}^{\mathrm{corr}}$	Corrected residual error after subtracting the estimated error from the raw NMEA error.

**Table 5:** Final 26 input features used by the random forest LOS projection error estimator.

No.	Feature	Category
1	Elevation angle	Geometry
2	Azimuth angle	Geometry
3	LOS East component	Geometry
4	LOS North component	Geometry
5	$C/N_{0}$	Signal strength
6	$\Delta C/N_{0}$	Signal variation
7	Relative CDD	Code-derived
8	Absolute relative CDD	Code-derived
9	SV time uncertainty	Uncertainty
10	Log SV time uncertainty	Uncertainty
11	Pseudorange-rate uncertainty	Uncertainty
12	Log pseudorange-rate uncertainty	Uncertainty
13	AGC	Hardware
14	State: code lock	State flag
15	State: bit sync	State flag
16	State: subframe sync	State flag
17	State: TOW decoded	State flag
18	State: millisecond ambiguity	State flag
19	Multipath indicator	Quality indicator
20	SNR	Signal quality
21	GNSS signal frequency	Frequency context
22	NMEA accuracy	NMEA uncertainty
23	Epoch mean $C/N_{0}$	Epoch context
24	Epoch standard deviation of $C/N_{0}$	Epoch context
25	Epoch mean SV time uncertainty	Epoch context
26	Epoch standard deviation of SV time uncertainty	Epoch context

**Table 6:** Feature correlation and RF importance analysis for the 26 selected features.

No.	Feature	Category	Corr.	|Corr.| Rank	RF Imp.	RF Rank
1	Elevation angle	Geometry	−0.0243	21	0.6299	12
2	Azimuth angle	Geometry	−0.0702	16	1.7599	2
3	LOS East component	Geometry	−0.0246	19	0.8587	7
4	LOS North component	Geometry	−0.1344	7	4.3882	1
5	$C/N_{0}$	Signal strength	−0.1487	4	0.8283	8
6	$\Delta C/N_{0}$	Signal strength	0.0034	24	0.4460	15
7	Relative CDD	Code-derived	−0.0135	23	0.5220	13
8	Abs. relative CDD	Code-derived	0.0430	17	0.4082	16
9	SV time uncertainty	Uncertainty	0.1371	6	0.7172	11
10	Log SV time uncertainty	Uncertainty	0.1328	8	0.0854	23
11	PR-rate uncertainty	Uncertainty	0.1485	5	0.7761	10
12	Log PR-rate uncertainty	Uncertainty	0.1798	2	0.2875	18
13	AGC	Hardware	0.0245	20	0.7829	9
14	State: code lock	State	−0.0822	13	0.1281	20
15	State: bit sync	State	0.1088	10	0.1229	21
16	State: subframe sync	State	0.0795	15	0.0895	22
17	State: TOW decoded	State	−0.0822	14	0.0000	24
18	State: msec ambiguity	State	−0.0844	12	0.1967	19
19	Multipath indicator	Quality indicator	–	25	0.0000	25
20	SNR	Signal strength	–	26	0.0000	26
21	GNSS signal frequency	Frequency	−0.0325	18	0.9772	6
22	NMEA accuracy	NMEA	0.1162	9	1.0457	5
23	Epoch mean $C/N_{0}$	Signal strength	−0.2351	1	1.2086	3
24	Epoch std. $C/N_{0}$	Signal strength	0.0236	22	1.1275	4
25	Epoch mean SV time unc.	Uncertainty	0.1748	3	0.2944	17
26	Epoch std. SV time unc.	Uncertainty	0.0852	11	0.4587	14

**Table 7:** Temporal WLS parameter-sensitivity results.

τ	*M*	All RMS	Suspect-Excl. RMS	Mean Session RMS	Median Session RMS
120	240	1.0346	0.4564	0.5668	0.4994
120	360	1.0322	0.4402	0.5633	0.4871
120	480	1.0320	0.4378	0.5629	0.4852
240	240	1.0339	0.4328	0.5590	0.4762
240	360	1.0312	0.4019	0.5525	0.4523
240	480	1.0310	0.3955	0.5512	0.4473
360	240	1.0340	0.4244	0.5563	0.4680
360	360	1.0315	0.3874	0.5487	0.4391
360	480	1.0315	0.3791	0.5470	0.4325
480	240	1.0340	0.4201	0.5549	0.4638
480	360	1.0318	0.3800	0.5467	0.4323
480	480	1.0320	0.3706	0.5448	0.4248
720	240	1.0341	0.4158	0.5536	0.4595
720	360	1.0323	0.3724	0.5447	0.4254
720	480	1.0327	0.3620	0.5427	0.4169

**Table 8:** Overall horizontal positioning performance.

Evaluation Set	Raw NMEA RMS (m)	RF y-WLS RMS (m)	Proposed Temporal WLS RMS (m)
All data	2.747	1.109	1.033
Suspect session excluded	2.867	0.694	0.362

**Table 9:** Per-session positioning performance. Session labels are anonymized to avoid exposing raw data filenames.

Session	Raw RMS (m)	RF y-WLS RMS (m)	Temporal WLS RMS (m)	Flag
S1	3.693	0.761	0.447	–
S2	0.375	0.098	0.061	–
S3	0.221	0.376	0.119	–
S4	2.523	1.596	1.647	Suspect reference
S5	4.211	1.009	0.596	–
S6	3.172	0.759	0.386	–

## Data Availability

The raw GNSS logs are not publicly available because they contain detailed spatiotemporal field-observation records, including time- and location-dependent measurement information and device-specific metadata. Due to privacy and site-identification concerns, the raw data cannot be shared publicly. Aggregated results and processed summary tables supporting the findings are included in the manuscript.
